# ResTech: innovative technologies for crisis resolution

**DOI:** 10.1057/s41261-021-00154-4

**Published:** 2021-04-03

**Authors:** Giuseppe Loiacono, Edoardo Rulli

**Affiliations:** 1 Single Resolution Board, 1000 Bruxelles, Belgium; 2 SciencesPo, 75007 Paris, France; 3 University of Rome Tor Vergata, 00133 Rome, Italy

**Keywords:** ResTech, Bank resolution, Innovation, Big data, Machine learning, C88, C89, G20, G38, O31, O32

## Abstract

The use of financial technologies (FinTech) by financial market participants fostered a discussion among public authorities on the use technologies for regulatory (RegTech) and supervisory (SupTech) purposes. This paper discusses the application of innovative technologies to crisis resolution (ResTech) and sets out its potential scope of application. ResTech is the application of technologies: i) to support the work of resolution authorities in developing resolution plans and in resolving financial firms; and ii) to allow financial firms to achieve regulatory compliance and better risk management in a more effective and automated manner. This paper also argues that the features and market dynamics of resolution differ from those of RegTech and SupTech: there is little market incentive for the private sector to foster innovation in the area of crisis resolution. The lack of private sector incentives to invest in R&D on how to resolve a firm’s crisis leaves the task to resolution authorities. In addition, resolution-technologies may support the identification of optimal liquidation strategy for small and medium-sized financial firms, by ensuring the maximisation of creditors’ proceeds out of the insolvency estate.

## Introduction

Financial firms are progressively embracing innovative technologies. This trend is not new [1], but the great financial crisis (2007/09) pushed the financial industry towards innovation [2], supporting a wider adoption of technology by financial firms [3]. Financial innovations are profoundly changing people’s approach to financial and banking services: the demand for real time performance of financial transactions, brokering, access to payment services is steadily increasing, and reached an unprecedented peak during the Covid-19 outbreak [4, 5].

Profit opportunities have driven the development of innovative technologies applied to finance, which at present is commonly known as financial technology (FinTech). As already noted in literature, finance is the most digitalised sector and this phaenomenon is quite old [2, 6]. However, FinTech is a recently identified area since only in 2017 the Financial Stability Board (FSB) defined the phaenomenon as the “technology-enabled innovation in financial services” [7]. Yet, there is no widely accepted definition in literature. That is because the notion of FinTech covers a wide spectrum of innovative, or innovatively provided, financial products and services e.g., digital retail payments, digital wallets, FinTech credit, robo-advisors, and digital currencies and their underlying technologies.

FinTech fostered the discussion among supervisory authorities on the use of similar technologies from a regulatory perspective (RegTech). The use of automated and innovative technology solutions for compliance and supervision emerged in the literature around the term RegTech [8], a concept that was first defined by the United Kingdom’s Financial Conduct Authority as the adoption of new technologies to better manage and facilitate the delivery of regulatory requirements [9]. Against this background, the application of technology to improve how supervisory authorities perform supervisory tasks has been defined supervisory technology (SupTech).

This paper argues that the underlying dynamics of the adoption of innovative technologies for regulatory and supervisory purposes are distinct from those of resolution. Supervisors generally follow the innovations developed by market participants. A similar logic does not apply to ailing firms, as in resolution there is little or no incentive for market participants to foster innovation. For this reason, resolution authorities may need to take the lead on developing and fostering the application of innovative technologies that can help handling crises in an efficient manner.

This paper discusses the application of innovative technologies that may support resolution authorities to resolve the crisis of financial firms. The set of innovative technologies that could enhance financial firm’s crisis resolution are referred in this paper as “resolution technologies” (ResTech) [10]. ResTech may support all areas of a financial firm’s crisis resolution, from resolution planning to setting and adapting related resolution requirements (such as TLAC and MREL), as well as in carrying out all activities related to the execution of resolution actions. These activities include the identification of the optimal combination of resolution tools to form the resolution strategy. In addition, ResTech may support the definition of the optimal liquidation strategy for small and medium size firms, by ensuring the maximisation of creditors’ proceeds out of the insolvency estate.

## Literature review

The adoption of innovative technologies by financial sector’s supervisors has been defined as Supervisory Technology (SupTech) [11]. SupTech mainly consists in the adoption of advanced data analytics and technologies to process large volumes of data potentially leading to, or enabling, real time supervision [12]. In addition, SupTech aims at reshaping the way regulations are drafted and adopted [13]. A recent Financial Stability Board report lists a variety of case studies and provides practical examples of deployment of RegTech and SupTech applications [14].

Innovative technologies underpinning RegTech and SupTech could also support the tasks performed by resolution authorities. Resolution is the restructuring of a financial firm by a resolution authority. Notably, resolution aims at orderly managing a financial firm’s failure safeguarding the public interests, preserving the continuity of critical functions, financial stability and, as opposed to public bailouts, minimising costs of taxpayers [15]. The resolution action consists in the use of one or more resolution tools by a public authority to manage the failure of financial firms in an orderly way. Resolution is an international concept, which may however cover sensibly distinct meanings across jurisdictions and legal orders. While the concept is generally linked to firms holding a banking licence, this paper refers to a wider set of financial firms. Special regimes for resolving crisis are in place, or in the process of being implemented for investment firms (such as brokers and dealers), insurance companies [16] and central clearing counterparties.

Before 2008 most countries lacked specialised legal frameworks and clearly defined lines of governmental-administrative responsibility for the resolution of failing banks and, in particular, of systemically important banks. The introduction of special resolution regimes [17] for large banks gained momentum at the outset of the 2007–2009 financial crisis [18, 19], whose defining characteristic was the public bailout of firms considered too big to fail [20]. Special resolution regimes targeted the issue, outlining the principle that shareholders and creditors, rather than taxpayers, should bear the cost of a financial institution crisis, thus introducing the concept of bail-in [21] as opposed to bailout [22]. In the following years, international standard setters [15, 23, 24], supranational regulators [25] and governments [26] have taken steps to reduce the impact of the failure of financial firms on public finances and related spill over effects [27].

A key element of resolution regimes is that banking groups shall hold at all times an adequate level of loss-absorbing and recapitalisation capacity [28] in order to support the implementation of a resolution strategy to resolve their crisis [29]. The FSB Key Attributes’ guiding principle is that firms must have an adequate level of loss-absorbing capacity to implement a resolution strategy that minimises any impact on financial stability, ensures the continuity of critical functions, and avoids exposing public funds to losses [15]. To achieve this, banks that are deemed to pose systemic risk at a global level (Globally Systemic Important Banks—G-SIBs) need to hold an amount of equity and loss absorbing debt, that the FSB defined Total Loss-Absorbing Capacity (TLAC) [30]. In the European Union (EU) the concept of loss absorbing capacity is extended to all banks, and is defined as the Minimum Requirement for own funds and Eligible Liabilities (MREL) [31].

Another key concept for the development of this paper is the resolution plan [32]. Even though resolution plans have different names across jurisdictions (e.g. living wills), they all refer to comprehensive documents, drafted by resolution authorities, which describe the individual features of a financial firm and detail how its potential crisis would be resolved [33]. Typically, resolution plans includes the preferred resolution strategy for a financial firm, and the resolution tools to apply.

### The European Union framework

In 2014, the EU introduced a special framework to deal with bank and financial firms crises, ruled by the Bank Recovery and Resolution Directive (BRRD) [34]. The BRRD sets out common rules to prevent crises and to resolve failing banks and investment firms in an orderly manner. The framework requires banks to prepare recovery plans, while resolution authorities are mandated with the drawing up of resolution plans, to be updated on an annual basis. Resolution authorities are also responsible to set the MREL, and for that reason, the legal framework empowers them to collect a wide set of data on a regular basis.

The EU framework provides resolution authorities with four resolution tools [35] for resolving failing banks:

- The *“sale of business”* tool, which allows resolution authorities to perform the full or partial transfer of a financial firm’s assets and liabilities to a buyer;
- The *“bridge bank”* tool, which follows a similar logic and allows that assets, liabilities and/or shares are transferred to a temporarily publicly owned financial firm;
- The *“asset separation”* tool, which grants the possibility to transfer the firms’ assets to an asset management vehicle; and
- The *“bail-in”* tool. The bail-in tool provides that the equity and debt of an ailing financial firm can be written down or converted, placing the burden on the shareholders and creditors of the firms itself, rather than on the taxpayers. The rationale of the bail-in tool is simple: in an firm’s resolution, shareholders are first in line to cover losses; the creditors are also asked to contribute by absorbing further losses or recapitalisation needs, moving from the most junior to the most senior layer of the creditor hierarchy in a given jurisdiction [36]. Under the EU framework, firms are required to hold at all times a minimum amount of own funds and loss absorbing debt to support an effective resolution strategy.

In the EU framework, the resolution regime was introduced to minimise negative repercussions of bank crises by preserving the critical functions of the resolved banks. For small and medium sized banks that do not perform functions that are critical to the real economy, there is no application of a special resolution regime. These banks remain subject to national insolvency laws, and their crisis management is normally aimed at maximising creditors’ returns (in some cases, these insolvency regimes give precedence to certain creditors over others: this is typically the case of protected depositors). While some European countries have developed bank-specific insolvency procedures, in others the insolvencies of small and medium banks are managed under the ordinary corporate regime [37].

### The United States framework

In the United States (US), a resolution regime for financial firms was introduced in Title II of the Dodd-Frank Act (DFA) in 2010 [38], which provides the Federal Deposit Insurance Corporation (FDIC) with the tools to resolve systemically important financial companies. However, the latter FDIC has been in charge of resolving failing deposit-taking entities since the adoption of FDIC Act in 1950 [39]. Deposit-taking firms are subject to a special regime that provides for both resolution and liquidation tools. The Federal Deposit Insurance Act (FDI Act) empowers the FDIC with resolution tools including a range of purchase and assumption transactions (P&As), a bridge bank tool and liquidation powers. The key feature of P&As is that the insured deposits of the failing bank are offered to a bidder, normally a healthy bank [40]. Where the failing bank’s assets are insufficient to cover the deposits transferred to the bidder, the latter may receive funding to cover the imbalance. Since 1991, the FDIC may only choose the least costly option between providing support to a suitable P&A transaction and directly paying off depositors [41]. In specific circumstances, the FDIC also has the power to form a new nationally chartered institution, known as a bridge bank.

The DFA sets out rules to manage the crisis of “financial companies”, a set of firms that includes large holding companies. Section 165(d) of Title I of the Dodd-Frank Act requires certain non-bank financial companies, and bank holding companies with total consolidated assets of $250 billion or more, to submit resolution plans periodically to the FDIC, the Federal Reserve Board, and the Financial Stability Oversight Council. One of the objective of the DFA is *“to provide the necessary authority to liquidate failing financial companies that pose a significant risk to the financial stability of the United States in a manner that mitigates such risk and minimizes moral hazard”* (DFA, s204). Interestingly, such authority needs to be exercised in a way that creditors and shareholders bear the losses of the financial company; this principle mirrors to the idea of “burden sharing” that underpins the EU bail-in tool.

### State of the art

The activity of resolution authorities is largely digitalised. Like supervisors, most resolution authorities perform their tasks using traditional digital solutions to draft, share and store documents. Off-site activities are the norm in resolution, with financial firms being subject to the obligation to provide documents, information and balance sheet data on a regular basis (reporting) and being subject to public disclosure obligations. Resolution authorities collect data and information to prepare a resolution plan, normally on an annual basis.

Data are collected in different formats [42]. Spread-sheet applications, such as Microsoft Office Excel, have been largely used for data collections in the last three decades. While regulators increasingly require the standardization of formulas using the eXtensible Business Reporting Language (XBRL) [43–45], Excel is still largely in use to ensure the consistency of reported data. Pure planning activities are generally performed using business software, such as widely known word processors providing for input, editing and output of text, e.g. Microsoft Word or Apple Page.

Data collections are generally based on in-house developed data warehouse. Large amount of structured data on resolution reporting flows periodically from financial firms to resolution authorities via excel, XBRL or word documents. Data are then typically stored in a central repository, the data warehouse. These structured data can be retrieved via fixed reports, with few possibilities for the business units to manipulate or create ad-hoc analyses.

In the context of resolution execution, authorities (or in certain jurisdictions liquidators appointed by either authorities or courts) need to receive updated data to perform the valuation of assets and liabilities. These data are needed to properly execute the resolution actions or to distribute the insolvency estate proceeds. Depending on the features of crisis resolution procedure, different objectives may be pursued, ranging from securing financial stability, protecting depositors or maximising the value for creditors [37, 46].

## The case for ResTech

Technology is shaping the financial services industry (FinTech). Recent history witnessed a tremendous increase of technology applications among financial market participants. Although in the past finance and technology have advanced together, following the 2007/09 financial crisis the speed of change and range of new entrants including FinTech and BigTech firms [46] in the financial sector have been remarkable.

If regulators sit on the fence, they may not keep the pace of the industry that drives the technological development. To mitigate the risk that only firms drive technological development, some regulators took the lead in the developments of financial technologies. This led to the rise of regulation technology or “RegTech”. The term was first introduced by the United Kingdom Chancellor, who mentioned it in the 2015 budget [48], promising to support *“*new technologies to facilitate the delivery of regulatory requirements*”* to the financial services sector.

RegTech refers to the use of technology in the context of regulatory monitoring and reporting, and includes enhanced compliance to the benefit of the finance industry [12]. More recently, the Bank for International Settlements (BIS) adopted a wider notion, defining RegTech as innovative technologies that can support financial institutions to comply with regulatory requirements and to pursue regulatory objectives [49]. Finally, recent studies [10, 50] developed RegTech in the supervisory context (SupTech).

Supervisors generally lag behind innovations developed by market participants. Internal ratings-based models are one tangible example of this phenomenon applied to credit risk management. Counterparties’ creditworthiness assessment is an evergreen challenge in banking. Thus, market participants developed sophisticated credit-scoring internal models, which have subsequently been endorsed by regulators [51].

This paper argues that the drivers for the adoption of technology for supervisory purposes are distinct from those of resolution. In resolution, there is little private sector incentives to invest in R&D on how to resolve a firm’s crisis; this task is therefore left to resolution authorities and regulators. One example from early 2010s is the introduction of bail-in, which, as opposed to public bailouts, limits banks’ moral hazard by ensuring that losses are first borne by their shareholders and creditors. The bail-in tool is an innovation in the resolution framework introduced by regulators; it avoids the consequences of a lengthy and complex judicial insolvency procedure [52] while preserving the critical function of a financial firm, an outcome on which a financial firm would normally not invest resources.

This paper defines ResTech as the innovative technologies which could: (i) support the work of resolution authorities in developing resolution plans and in resolving financial firms; and (ii) allow financial firms to achieve regulatory compliance and better risk management in a more effective and automated manner. This paper identifies four main areas of ResTech:

(i) Technology supporting resolution planning activities. This entails the use of automated resolution planning and innovative data collections and processing technologies.
(ii) Technology supporting the execution of resolution actions. It includes the adoption of innovative technology supporting the definition of the optimal resolution strategy and resolution tools, including the orderly wind down.
(iii) Technology supporting the cross-border exchange of information to coordinate global resolution actions.
(iv) Technology supporting financial firms’ cooperation with resolution authorities, which may facilitate the transition of financial firms to automated compliance for resolution purposes.

**Fig. 1:**
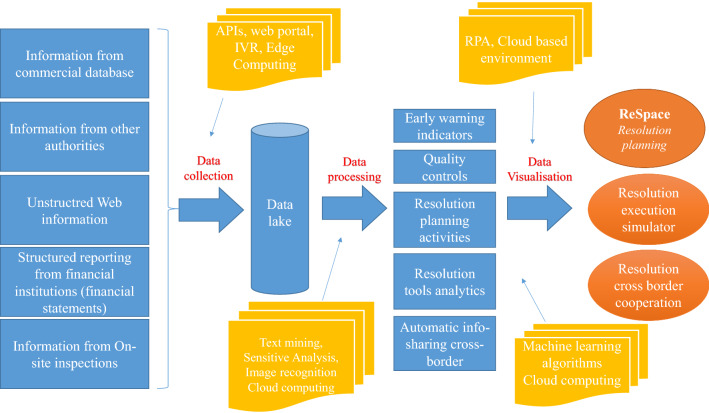
The ResTech framework

ResTech would rely on big data technologies, which can be generally defined as software-utilities designed to analyse, process and extract the information from an extremely complex and large data set which the traditional data processing software could not deal with. Big data technologies assist users in different phases, from data collection to data processing and visualisation. The collection phase would consist in making use of big data technologies to gather resolution reporting data from different sources and store them in a data lake. Cloud computing, text mining applications and machine learning algorithms may allow users to process such data for resolution purposes. Finally, the data visualisation phase allows to explore and visualise them in a dynamic and digitalised environment (Fig. 1).

The following sections illustrates in detail potential applications of resolution technologies in resolution planning, execution and cooperation with other authorities.

## Uses of ResTech in resolution planning

The result of resolution planning is the resolution plan. The resolution plan is usually a text document that includes a holistic analysis of the optimal strategy to resolve a crisis in any given time, including the determination of the level of the loss absorbing and recapitalisation capacity needed to apply burden-sharing measures, such as the bail-in tool. Resolution plans are in most jurisdictions developed by the resolution authority based on a wide range of quantitative and qualitative information coming from both the financial firms concerned and from financial sector authorities. A ResTech-driven work environment could support resolution planning to become more dynamic by transforming the resolution plan from a text document to a digitalised cloud environment.

**Fig. 2:**
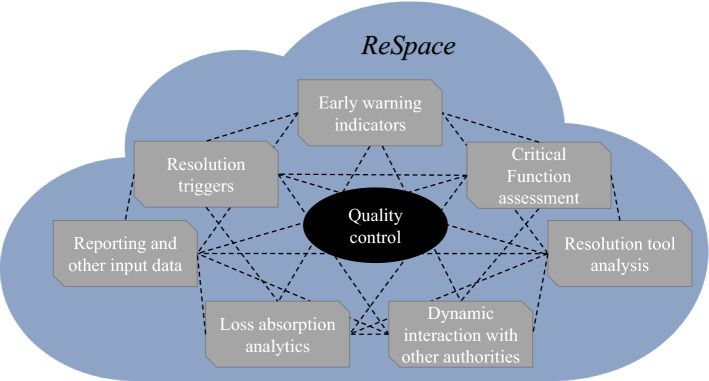
ReSpace: simplified visual

Resolution authorities may adopt a resolution cloud-based platform for the activities of resolution planning, making use of cloud computing, edge computing and streaming analytics technologies. This paper supports exploring available technologies to set up a single resolution virtual workspace (hereinafter, “ReSpace”) to enhance resolution planning. Resolution planning input data would regularly flow from authorities and from financial firms to the resolution authority’s data lakes and could be automatically consolidated in ReSpace (Fig. 2).

ReSpace would allow real-time consolidation and update of planning input files. Consolidation involves the integration of data from multiple sources and formats. Data sets often contain relevant information about different dimensions of the same firm (e.g. balance sheet data, qualitative information, management-information systems), and manually integrating them can be a time-consuming task prone to errors. With enhanced visualisation tools, such as what this paper defines ReSpace, automated data consolidation in cloud computing services would reduce time inefficiencies and eliminate manual errors.

The resolution plan could ultimately become a living document. Each authority involved in resolution planning would have its own access to the relevant part of the resolution plan in a dynamic environment. Similarly, the financial firm itself may be granted access rights to certain information contained in ReSpace. Limitations could be envisaged to cater for confidentiality and privacy. Assuming that all the resolution plan’s underlying information are gathered in ReSpace, the resolution plan itself would become a virtual tool, where all chapters would be dedicated ReSpace pages, automatically linked to the underlying data, the applicable legal framework and resolution policies.

Featuring enhanced visualisation techniques, the common workspace we name ReSpace could be seen as a user-friendly resolution platform. This would include visualisation interfaces that would benefit from big data architectures to provide seamless and interactive user experience with minimal latency. This common virtual working space may serve as a resolution dashboard simplifying the experience of using data and providing resolution plan contributors with “at-a-glance” visibility of the status of the resolution plan, linked to the underlying fundamentals of the financial firm. Eventually, ReSpace would substitute static text documents as well as spreadsheet-generated dashboards that require manual updating. To extract the most meaningful insights from data, the applications and the architecture underpinning ReSpace could allow for numerous analytical operations, such as drilling up (i.e. summarising data along one dimension) and drilling down (i.e. navigating deeper along a dimension), as well as slicing, dicing, pivoting and overlaying data across multiple dimensions.

### Reporting

The inputs to a resolution plan are massive and heterogeneous. They include data from commercial databases, data from other resolution authorities, unstructured web information, regular reporting from financial firms and occasional reporting from on-site inspections. Generally, public authorities’ statistical frameworks rely on data warehouses to aggregate and store information. Data warehouses are database resulting in highly structured data model designed for reporting. They contain quantitative metrics and the attributes that describe them. In resolution reporting, for example, data warehouse is used to store financial firms’ balance sheet data. Typically, unstructured information sourced from web or qualitative information are excluded from data warehouses.

Resolution authorities may progress from a data warehouse to a data lake approach, which is the most recent evolution of a data warehouse. Unlike the latter, which only retain structured data, data lakes can store in a unique environment raw copies of all source system data and transformed data used for tasks such as reporting, visualization, advanced analytics and machine learning. Raw data can include structured data from relational databases (rows and columns), semi-structured data (CSV, logs, XML, JSON), unstructured data (e-mails, documents, PDFs) and binary data (images, audio and video).

All types of inputs to a resolution plan may be stored in data lakes, independently from whether the information is qualitative or quantitative, structured or unstructured and, importantly, regardless of whether the information in question would be used or not, remaining at disposal of users. In data warehouse, users get reports in structured way (typically spreadsheets) which are then extracted in fixed outputs (with limited possibilities of ad-hoc extractions). These outputs can then be shared within the organisation, and serve as input for further analysis. With data lakes, users can go to the lake and work with the very large and varied data sets they need; there is no need to extract the data to another environment to perform, for instance, analytical calculus.

Regarding data processing, Application Programming Interfaces (API) are the big data innovative solutions at disposal of resolution authorities. API is an interface which defines interactions between multiple software intermediaries, and that allow organise bulks of data and information [53]. API and machine learning algorithms operated on data lake would facilitate the reconciliation of data and simplify the valuation of assets and liabilities. Resolution reporting data could be gathered from different authorities in different jurisdictions, superseding any criticality stemming from the different databases, thus supporting and enhancing compliance. An important feature of API for resolution planning is that it allows databases to connect and communicate to each other. For example, a resolution authority’s data lake could “call” the bank’s database and download certain data needed to update the resolution plan in real time. Alternatively, the bank could automatically “call” the resolution authorities’ data lake to check if there is any new decision or compliance document and, if so, download it. A practical application of APIs for enhanced compliance is the following: with API systems financial firms’ public disclosures on resolution requirements (for example, the level of MREL or TLAC) could be immediately matched with the applicable resolution policy, to enable the resolution authority to automate the verification of the information disclosed to the public.

Finally, machine-learning algorithms could improve data reconciliation and mitigate timing issues while reducing reporting costs. As an example, machine-learning algorithms could automatically reconcile data coming from regular reporting with market data and data from banks’ on-site inspection in data lakes, thus eliminating the risk and the costs stemming from resubmissions and data reconciliations.

### Early warnings for crisis detection and resolution triggers

Several information from the web or the balance sheet may be processed to anticipate or predict the crisis of individual firms. In particular, big data analytics applied in resolution planning can predict pre-insolvency and insolvency scenarios. A great number of financial health indicators can be assessed against unstructured information available online, thus allowing supervisors and resolution authorities to predict the evolution of a financial firm balance sheet. Unstructured information may be gathered from a wide range of sources, including businesses and social media. With the advent of the internet and mobile technologies, social media have become an integral part of our society. Social media platforms such as blogs, discussion forums, review sites and social networks can be used to easily create and share the so-called user-generated contents [54], which contain valuable information to be analysed. Any regular reporting and on-site collected data could thus be complemented and enriched by data collected from text mining applications covering social media, banks disclosures, financial newspapers, and financial database.

Resolution authorities can make use of text mining algorithms and web scraping technologies to develop sentiment analysis for predicting crisis cases. Text mining is a technique to exploit information embedded in textual documents. It is used to monitor the web and extract relevant information, and transforming them from unstructured to structured data that could be analysed and used to inform business decisions [55]. In particular, web-based information coupled with data coming from commercial databases and regulatory reporting, could be used to develop early warning indicators for crises detections. With Robotic Process Automation (RPA) systems early warnings indicators would be automatically linked to the underlying data, updated and visualised in ReSpace to inform experts in their resolution planning activities.

ResTech solutions could support the determination of the optimal timing of resolution. The benefits of resolution actions are maximised if resolution authorities are ready to escalate from resolution planning to resolution execution at the appropriate timing. A number of jurisdictions introduced the idea that financial firms, and banks in particular, should enter resolution before they are insolvent, i.e. when they are likely to fail, as anticipated insolvency would avoid disruption and loss of value. Applied to banks, this means that the resolution authority needs to timely identify when they infringe, or are likely to infringe in the near future, the requirements for the banking licence or other type of requirements for continuing authorisation.

The triggers of resolution in most jurisdictions are a combination of qualitative and quantitative indicators signalling that the financial firm’s condition of failure may materialise in the near future. The quantitative resolution indicators defined by the resolution authority’s staff would be continuously updated with intra-day data inputs, supporting the experts’ analytical judgment that remains crucial to perform a holistic quantitative and qualitative assessment.

### Loss absorption analytics

Streaming analytics are a type of real-time data analysis that allows recording and performing simple calculations on large amounts of constantly updated data. This solution accepts inputs from multiple sources, process them immediately and return insights. Streaming analytics generally allow users to monitor real time data streams and respond to complex conditions within milliseconds.

Streaming analytics models can be used to support the calibration financial firm’s loss absorbing capacity (MREL, TLAC). Resolution plans normally indicate the loss absorbing capacity that needs to support a timely and efficient resolution. Streaming analytics may support the calibration of loss-absorbing resolution requirements to bank specific business models and resolution strategies.

In addition, resolution authorities may make use of machine learning predictive analytics. Predictive analytics is the area of the advanced analytics used to make predictions about future events. Predictive analytics leverages on a number of techniques that include data mining, statistics and machine learning to analyse actual data to make predictions. These techniques may be used in particular to anticipate future loss-absorbing needs, or to design dynamic requirements that would be adjusted automatically to market conditions.

### Quality control

A common working space, such as the one we defined above (ReSpace), could also enhance quality control processes. A visualisation tool would facilitate comparison of resolution plans across different banks, alerting users on a misapplication of laws and/or policies, yielding different outcomes for the same situation. In addition, this would allow experts to go into a much greater level of detail and derive deeper insights on certain risk indicators. The adoption of ReSpace by resolution authorities competent for banking groups active in more than one jurisdiction could facilitate the identification of inconsistencies, for instance by fostering the mutual understanding on what are the functions of a bank that need to be considered critical, and may allow authorities to verify the adequate calibration of the level of loss absorbing capacity for each firm.

### Distributed ledger technology for resolution planning

Distributed Ledger Technologies (DLT) are information and data recording tools developed on a decentralised network. Information are stored and maintained on a distributed ledger, i.e. a repeated digital copy of data at multiple locations, as in block chain. These technologies enable nodes in a network to securely propose, validate, and record a full history, state changes (or updates) to a synchronised ledger that is distributed across the network’s nodes. DLT applied to resolution planning can improve data governance and security. Concretely, where a resolution plan would be distributed across nodes of the chain, it would be possible for resolution authorities to implement an automatic validation system through a consensus algorithm that replicates, shares and synchronises digital data across different locations. This technology may enhance the cooperation among authorities involved in resolution planning (namely, resolution authorities and supervisors, but also, AML authorities, Ministries etc.). Also, where the decision making process requires different resolution authorities to jointly draft a resolution plan, as may be the case in cross-border planning, DLT technologies may help fostering cooperation and trust. The unique feature of a blockchain database is that once data has been entered, they become part of the chain and cannot normally be deleted. This makes them a highly secure environment.

## Uses of ResTech in resolution execution

The execution of a resolution action relies on a strategy influenced by multiple factors. The resolution plan sets the scene for the execution of resolution actions; resolution planning and execution are complementary and there is a continuum between the two. When the decision to resolve a firm is taken, resolution authorities must swiftly move from the planning to the execution phase, determining the resolution strategy and tools to apply on the basis on the resolution plan.

In order to be effective, any resolution action must be implemented swiftly and preferably during market closure. For this reason, often authorities refer to the “resolution weekend” to indicate the timeframe in which resolution actions should be performed. Any resolution action, which is prolonged overtime, would be likely to achieve less optimal results than a swift resolution. Time inefficiencies increase the risk of further financial deterioration of the firm’s financial position, for example through bank runs, share buybacks and debt early redemptions.

ResTech could complement and boost the efficacy of existing resolution tools. Existing crisis management and resolution frameworks rely on tools developed by the industry and regulators before the introduction of the innovative technologies discussed in this paper. ResTech would not replace existing resolution tools, whose use and efficacy have been empirically demonstrated. Rather, technology supporting resolution would complement existing resolution tools and boost their efficacy.

**Fig. 3:**
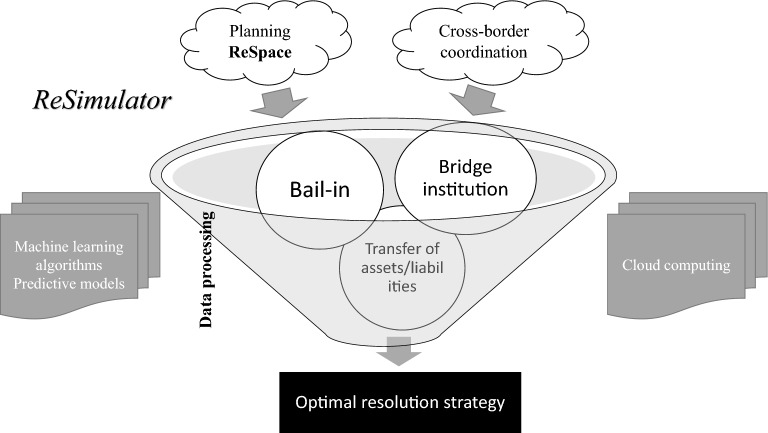
A resolution simulator: simplified visual

One example could be the adoption of a resolution simulator, intended as an analytical and visualisation tool. A resolution simulator would support the identification of the tools to apply and the implementation of resolution actions. It would inform the resolution authorities of the optimal combination of resolution tools to form the resolution strategy, in a way that ensures meeting resolution objectives maximising its positive effects to financial stability. A resolution simulator based on machine learning and artificial intelligence would process—at any time—all the information available in the planning phase (for example, in ReSpace) to identify and allow the prompt adaptation of the best actions and tools to manage the financial firm crisis in an orderly way (Fig. 3).

Resolution objectives can be better achieved by simulating the outcome of the application of technology-enhanced resolution tools. The optimal resolution strategy would be composed of a combination of enhanced resolution tools, complemented by the analysis on the potential liquidity needs to finance the resolution actions, and based on the applicable national governing laws. Assessing the need of liquid funds in resolution is key to the successful implementation of a resolution strategy. Machine learning algorithms may be used to predict the liquidity needs in a given resolution case, e.g. the cash needed to pay off the firms’ debts falling due during resolution. The following paragraphs describe how the adoption of ResTech can enhance the efficacy of existing resolution tools.

### Transfer of assets and liabilities

Transfer tools are among the most common resolution tools in a number of jurisdictions that implemented the FSB provisions for an effective resolution regime. These involve the full or partial transfer of assets and liabilities of an ailing firm to an acquirer. Purchase and assumption transactions may be complemented by the bail-in tool, but can also be accompanied by the setting up of an asset management vehicle for the disposal of the financial firm’s non-performing assets; thus transferring to the identified acquirer only the good part of the resolved financial firm.

Machine learning predictive analysis could support resolution authorities in the application of a transfer tool. This support can cover any aspect of the process, starting from the set-up of virtual spaces where machine-learning algorithms would identify the most suitable acquirers based on potential synergies between the two businesses, available liquidity, and forecasted profits of the entity resulting from the potential asset transfer. Some limited resolution planning information and their visualisation may be made available to a set of potential purchasers, thus replacing the existing facilities serving valuation purposes, where generally documents and information for performing due diligence are uploaded.

### Bridge institution

The bridge institution tool concerns the creation of a publicly owned new financial firm (normally a licenced bank), to which assets and liabilities are transferred allowing the continuation of critical functions. The more assets are transferred, the higher the recapitalisation needs. The success of the bridge institution tool depends on distinct factors. On the one hand, the availability of loss absorbing capacity in the failing entity that allows that losses are absorbed by the shareholders and creditors of the resolved firm. On the other hand, the punctual determination of the perimeter of assets and liabilities linked to critical functions that need to be swiftly transferred to the bridge institution. The latter typically represents a difficult and resource intensive task in the resolution planning phase.

Machine learning algorithms may support the creation of a bridge institution, by predicting the optimal perimeter of assets and liabilities linked to critical functions to be transferred, and the perimeter of non-core assets to be left in the legacy entity. The simulation of loss absorption capacity coupled with forecasts on perspective earning of the newly created bridge institution would inform experts on the feasibility and on the rate of success of similar resolution cases.

### Bail-in

The bail-in tool constitutes one of the real innovations brought by rule makers in the context of resolution. As discussed above, in a crisis, the bail-in tool allows the cancellation of equity and debts to absorb losses and the conversion of debt into capital for recapitalisation. Its application has been limited due to its severe consequence it has on creditors, and ultimately on financial stability. In resolution, it is the most intrusive tool as creditors suffer losses; however, it is also probably the most effective tool as it allows immediate loss absorption with existing creditors and shareholders. Typically, complex and large banks, for which no third–party purchaser could be found, are expected to be resolved with the bail-in tool.

The application of the bail-in tool is generally complex and require granular data on liabilities’ classes. In addition, where special resolution regimes for financial firms differ from ordinary insolvency ones, the bail-in tool application is safeguarded by the No Creditor Worse Off Principle (NCWO). The FSB states that resolution powers, and in particular bail-in, should be exercised “in a way that respects the hierarchy of claims while providing flexibility to depart from the general principle of equal (*pari passu*) treatment of creditors of the same class, with transparency about the reasons for such departures, if necessary to contain the potential systemic impact of a firm’s failure or to maximise the value for the benefit of all creditors as a whole” [15]*.* Any deviation from this principle, i.e. any losses incurred in resolution higher than in liquidation, could result in a compensation to creditors.

ResTech could enhance the way bail-in is applied. A key analysis underpinning the application of the bail-in tool is the determination of a firm’s loss absorbing and recapitalisation needs. By leveraging on a set of information aggregated in a data lake and processed with machine-learning algorithms, it could be possible to simulate the application of the bail-in tool forecasting its effects on each class of creditors in the hierarchy of claims, and comparing the losses incurred in resolution with those hypothetically incurred if the financial firm were to be liquidated. The result would thus be the determination of the limit to the application of the bail-in tool without breaching the NCWO principle. All the operational aspects of the bail-in tool can be automated using streaming analytics (for example, de-listing from trading bank’s securities, or relisting new instruments of ownership following resolution).

Finally, text-mining applications adopted by resolution authorities and financial institutions could be applied to scan thousands of banks’ contracts to recognise the presence of bail-in clauses in contracts governed by the laws of other jurisdictions, and automatically categorise them by hierarchy of claim. Such data could be stored at the financial institution’s data lake to be retrieved promptly the resolution authority’s data lake, through APIs, to allow the swift execution of any resolution actions.

## Orderly wind down

Entities whose resolution strategy foresees liquidation under ordinary insolvency proceedings would not survive their crisis. These entities thus require resolution authorities to draw up a resolution plan aimed at maximising the safeguards of creditors’ rights and claims. That is because these entities do not perform critical functions, and their failure is assumed not to be likely the cause of disruption to financial stability. Therefore, liquidation entities are not resolved with the application of special resolution regimes, and their market exit is generally the solution that best meet the public interest.

These entities have even less incentive in investing resources for their “liquidation plan”. Thus, where resolution authorities develop technologies that can be used in the context of resolution, small and medium sized firms may benefit from the application of such technologies for their liquidation.

Machine learning and big data technologies can certainly improve the planning for orderly wind down. This paper suggests that resolution authorities are called on to adopt big data technologies which can then be made available to those entities willing to automate certain processes, such as auto valuation, real time balance sheet dashboards, risk indicators, early warnings and failure’s assessment tools. ResTech technologies can thus support authorities to achieve both the objective of resolution, such as safeguarding critical functions, and those of orderly wind-down, such as the maximisation of creditors’ proceeds.

As detailed in the previous chapter, machine learning algorithms can support the sale of business, as well as the identification of non-performing assets that can be disposed via asset management vehicles. These are already realistic use cases of ResTech that can support the execution of resolution strategies, but may also support the liquidation scenario. Notably, reference is made to technologies supporting the valuation phase and the assessment of applicable legislation and policies to guide firms’ liquidators. Early valuations based on machine learning tools can support authorities in performing crucial assessments such as the least cost test, which is a feature of the US resolution regime and also mentioned in the EU framework in Article 11 of the Directive on deposit guarantee schemes (Directive 2014/49/EU of the European Parliament and of the Council of 16 April 2014 on deposit guarantee schemes).

ResTech may also support the work of appointed liquidators, both in judicial and administrative proceedings. A financial firm’s liquidator needs to act quickly to determine impact of the failure and a plan for liquidating the estate. Big data technologies can provide solutions to the following insolvency phases:

(i) the real time assessment of creditors’ claims;
(ii) the placing of creditors’ claims along jurisdiction’s insolvency hierarchy; and
(iii) the simulation of the final distribution of proceeds based on technologies anticipating the outcome of lengthy judicial or administrative proceedings.

The adoption of ResTech for liquidation entities would allow the swift execution of the above mentioned liquidation actions and better maximise the value for creditors compared to traditional insolvency proceedings. Big data technologies may shorten the duration of liquidation procedures, thus saving costs for the financial firm’s insolvency estate, for example in terms of reduced procedural fees and lower interest accruing in favour of creditors. For example, machine-learning algorithms could be applied on insolvency databases that contain data on past insolvency cases to develop future optimal liquidation strategies.

## ResTech for financial institutions

The adoption of big data technologies by resolution authorities may support the transition of financial firms to automated compliance for resolution purposes. In the resolution context, the provision of information for resolution planning and crisis management is a key aspect. To this purpose, financial firms are often required by law to manage efficient and effective information sharing system with the use of state-of-the-art technologies to ensure financial firms accomplish this objective. In this respect, financial firms may ultimately benefit from the use of RegTech and SupTech [8] applications for compliance purposes in the resolution regulatory framework.

One example of RegTech for financial firms is automated reporting. Automated reporting enhances firms’ efficiency, avoiding that firms’ employees manually submit files (template-based reports) that take up a substantial amount of resources and are prone to errors [57]. Ad-hoc data requests from resolution authorities would not be done anymore via basic web portals, or via e-mail, but via the analytical technology that allows continuous information flows. At present, a number of financial firms have already started developing automated tools for reporting to supervisors [58]. This means that they may leverage the existing infrastructure and achieve the desirable automation in the context of resolution with limited additional costs.

One step further is the adoption of automated reading and machine learning technologies allowing financial firms to automate compliance. Financial firms could adopt automated compliance systems [59] and reduce staff in compliance services by disposing of technologies that read any regulatory document, from actual regulations to individual authorities’ decisions, and then automatically verify compliance, for example with MREL and TLAC requirements.

On a broader view, machine-readable solutions (e.g. regulatory radars [60]) automatically scan the legislation, official communications and authorities’ website to capture the development of new regulations. Regulatory radars could identify any issues of interpretation for closer examination by internal or external legal consultants. Such automation, using regular information flow, would reduce the on-site inspections to the minimum necessary. To this end, APIs can support the flow of information and the transfer of large volumes of data directly between the firms and the authority data lakes in an automated way, thus reducing general compliance costs, within the limit that ResTech could not replace expert judgments.

Box: ResTech and Anti-Money Laundering

ResTech can bridge the gap between resolution regimes and Anti Money Laundering (AML) policies. Although it might not seem obvious at first glance, the link between resolution regimes and (AML) is profound. Where a financial firm performs an illegal activity, it is exposed to a sudden failure, which can trigger severe disruption for a wide range of unaware stakeholders, and may affect the continuation of the firm’s critical functions. Hence, further to supervisors, resolution authorities could have an interest in developing innovative technologies that allow for the steady cooperation with AML authorities.

ResTech tools such as streaming analytics and DLT-based technologies could be used for example to identify real time suspicious transactions performed via financial firms. The need to bridge the gaps between AML and supervision [61], with an eye to the resolution of the financial firms involved in money laundering, has already been highlighted in literature [62]. Policy makers might consider taking into consideration bridging the gap between AML regulations and resolution regimes, by favouring the adoption of innovative technologies that put the two domains into communication. Recent FSB efforts in this direction seem to have already paved the way for including resolution regimes in the discussion on AI-based risk management for AML [7].

## Domestic and international cooperation: towards international standards setting?

The adoption of innovative technologies could achieve better results in a technologically neutral environment. The term “technological neutrality” is a principle of good regulation, which has distinct meanings, ranging from the “technical standards based on the results they aim to achieve”, to “limit to the use of regulations to orient market structures” [63]. In this paper, technological neutrality means designing technology in a way that makes it interoperable with the systems of other authorities.

RegTech, SupTech and ultimately ResTech can enhance domestic and cross-border cooperation between authorities. Interoperable technologies would enable supervisors, resolution authorities and authorities vested with oversight functions, including antitrust and anti-money laundering authorities, to cooperate in a timely manner. Theoretically, this holds true both in a domestic and in a cross-border environment. This is confirmed by recent studies [50, 64], which acknowledge that the adoption of technologies may enhance the mutual understanding in home/host relations [65]. In practice, the adoption of technologies increasing the efficiency of resolution actions is, at present, more likely to achieve these results in a domestic rather than in a cross-border context.

In a cross-border context, ResTech may help addressing some of the complex issues posed by the resolution of financial groups operating in various jurisdictions. Yet, ResTech is not, alone, the solution, as cross-border actions can only become safer and more efficient if the authorities involved in a cross-border resolution case have previously agreed on all other potential aspects concerning the resolution process.

For instance, where two or more authorities have agreed to share the data belonging to a financial group active in their respective jurisdictions, and once these authorities are confident that no data leakage would affect their work, ResTech could play a role in enhancing cross-border planning and, potentially, cross-border resolution. Innovative technologies applied to resolution planning and execution—as described above—could improve, and make safer, the data sharing for the preparation of resolution plans for cross-border financial groups and allow coordinated cross-border decision-making where groups with operations in distinct jurisdictions are failing. The drawing up of resolution plans for groups operating cross-border and the execution of resolution actions in different jurisdictions could leverage on algorithms that take into account both the economic reality of the financial firms concerned and the legal frameworks in which the execution of globally coordinated resolution strategy would take place. API and machine learning algorithms operated on data lake may facilitate data sharing and reconciliation by different authorities.

To achieve these results, ResTech requires a common understanding and an international taxonomy. Standard setters may play an important role in designing a common taxonomy [66] and a minimum set of standard for the adoption of technologies by resolution authorities, and put forward ideas to adapt existing financial rules to a digitalised environment [67]. Resolution authorities worldwide need to converge to respond the following questions: what technologies can be used to support resolution? How should resolution authorities define and make use of them in a coordinated manner? The answer to these questions should also clarify the need for technological neutrality. Even though we are not yet there, going forward, all these elements can help further facilitating authorities’ mutual understanding across jurisdictions [68].

ResTech can also enhance the full and effective participation of different authorities to international crisis-management fora. For instance, in the banking field, the use of neutral technologies, such as machine learning and text mining, may characterize the firm-specific agreements related to a cross-border Crisis Management Group (CMG). Normally, CMGs work based on non-binding agreements between the authorities supervising the companies belonging to a global systemically important banking group. In the future, these agreements could themselves be translated into technological applications, or leverage on technologies upon which authorities reach consensus, as it is increasingly happening to agreement in the private sector, which begin to be partly of fully automated (smart contracts) [69, 70].

In practice the resolution of cross-border financial institutions, in particular the systemically important ones, could be extremely complex for a variety of factors of legal, economic and political nature. The authors do not aim to present ResTech as the panacea for cross-border global cooperation in the area of resolution. Yet, the adoption of common technologies, if properly complemented with multilateral agreements, along with the adoption of converging standards in general resolution practices, may pave the way for closer cooperation among resolution authorities in the medium to long term horizon.

## Risks and challenges of ResTech

The benefits of ResTech need to be measured against the increased risks taken by resolution authorities. In a digitalised environment, resolution authorities would need to set up IT infrastructures and e-governance processes that prevent operational, cyber, reputational and legal risks. Resolution authorities relying on innovative applications need also to take up the challenges of recruiting big data scientist to cooperate with other crisis management and resolution professionals, and of setting up adequate internal control systems to prevent wrongdoings.

### “Black-box effect” risk

A fully-fledged “ResTech toolbox” driven by big data technologies may produce the black-box effect [71]. In general, complex and automated work environments raise concerns as to the lack of transparency around the way data are processed. If the underlying methodologies governing decision-making processes are not adequately disclosed to the market, information asymmetries could grow larger and financial firms may perceive that the decisions imposed to them by authorities are not replicable and that there is no control over decision-making. The opacity around certain technologies and IT applications could hinder the work of specialised audit staff and give ground to financial firms to challenge resolution authority’s decisions [72].

### Operational risks

Operational risks arise in resolution authorities when using automated technologies. If third party providers handle algorithms—as it is likely to happen in the case of regulatory authorities—data security, confidentiality and integrity must be ensured, in particular in the execution of resolution. Cyber-attacks are key threats in an automated environment, where the threats include data losses and interruption of supervisory activities.

In a ResTech environment, automation reduces the risks of manual errors; however, errors may derive from the improper set up of applications. For example, incorrectly calibrated algorithms regarding the simulation of the outcome of resolution actions might lead to the selection of a sub-optimal resolution strategy, posing risks to the overall efficacy of resolution actions. In this regard, ResTech would require authorities to invest in IT infrastructure for enhanced risk-management and internal control processes [10].

### Legal risks

The use of data-driven technologies exposes resolution authorities to increased privacy and business confidentiality risks. Typically, resolution plans contain sensitive information on financial firms and their managers. In addition, as they describe the strategy to resolve a financial firm’s crisis, they contain information that may become sensitive in a dynamic environment, e.g. the information on the resolution tools to be used at the moment of distress. The reduction of legal risks requires strong data governance and secure IT infrastructures.

Technology applied to resolution actions may pose issues to the allocation of liabilities stemming from technological applications. Similar to any technology-driven action taken by authorities and firms, technology applied to resolution activities can lead to mistakes and errors. Authorities might then be at risk of breaching regulations exposing them to potential liabilities towards stakeholders [73].

Liabilities stemming from the application of technology may be difficult to allocate. Where a resolution decision taken on the basis of a technological application—such as an algorithm—damages a third party, the question arises as to whether is the resolution authority or the technology provider that needs to be held liable for decisions guided by a technology.

Technologies developed in-house by resolution authorities would enhance data collection, storage and visualisation and usage (support to resolution planning and execution) and at the same time diminish the risk of legal risk stemming from third party providers mistakes or wrongdoings.

### Human resources

Human resources are key to assess and interpret results of ResTech outputs, as well as ensuring accuracy and completeness. Since technologies supporting resolution leverage on big data and algorithm-based processes, their adoption requires authorities to employ adequately skilled human resources, especially to address the “black-box” risks on algorithm-based results. The set of human skills that resolution authority may need to hire is broad and covers IT engineering and resolution-specific skills. For example, to understand how the resolution simulations forecast the optimal combination of resolution tools, the staff of resolution authorities—who most likely have legal or economics background—needs to be paired with data analysts.

## Conclusions

Regulators and resolution authorities have developed resolution tools with the support of existing traditional technologies. Resolution is the use of one or more resolution tools by a public authority to manage the failure of financial firms in an orderly way. The increasing use of financial technologies (FinTech) by market participants fostered the discussion among supervisors on the use of innovative technologies on the regulatory side (RegTech), and in its application in the context of the supervision of financial firms (SupTech). The resolution context is however peculiar: there is little market incentive for the private sector to foster innovation in this area, as resolution is a task of the resolution authority and is not a profit-making activity.

This paper suggests that technologies supporting resolution are developed by resolution authorities. To this end, this paper defines ResTech as the innovative technologies which could: (i) support the work of resolution authorities in developing resolution plans and in resolving financial firms; and (ii) allow financial firms to achieve regulatory compliance and better risk management in a more effective and automated manner. Technologies could be embraced by resolution authorities to automate certain resolution planning activities as well as to support the identification and the execution of the optimal resolution strategy. For global financial firms, ResTech could enhance further cross-border cooperation in resolution matters.

The ResTech toolbox would rely on state-of-the-art big data architecture and technologies. One example discussed in this paper is the shift from data warehouse to data lakes, which enable the gathering of structured (reporting) and unstructured (web-based) information. Data could be then processed in cloud computing environments with API solutions, among others. The use of machine-learning algorithms could support the analytical work behind the definition of early warning indicators and the identification of the optimal resolution strategy.

ResTech includes visualization products for resolution planning and execution activities (such as dashboards, automated reports etc.). The resolution plan would become a digital tool in a cloud-based work environment that this paper defines ReSpace. Data would regularly flow from authorities and firms to the resolution authority’s data lake and be consolidated in ReSpace, with automated quality checks. Users may visualise the input data underlying each planning activity and any applicable law provisions and policies.

ResTech would eventually introduce a continuum between resolution planning and execution. A resolution authority that avails itself of ResTech might reduce the distance between the ex-ante description of the actions that would be taken in a crisis scenario as detailed in the resolution plan and the execution of the resolution strategy. Resolution plans would benefit from real-time updates and users’ expert judgments. Resolution simulators could provide resolution experts and with the optimal combination of resolution tools. In a ResTech-framework, the difference between planned resolution actions and the actions that are actually taken can be reduced up to zero.

In addition, ResTech may also support the planning for optimal liquidation strategies for small and medium-sized financial firms. These entities are less likely to invest in R&D, but their disruptive failure may entail risks that could be handled effectively with the adoption of innovative technologies. ResTech for small and medium-sized financial firms may support achieving the maximisation of creditors’ proceeds out of the insolvency estate.

Notwithstanding the promising aspects of ResTech, its adoption by resolution authorities might not be the answer to all existing challenges. Further multidisciplinary research is required to assess the benefits of technology supporting resolution against its risks, ranging from the black-box effect risk to the challenge of recruiting staff with expertise in both resolution framework and big data technologies.

Going forward, relevant actors and stakeholders would need to develop a common taxonomy for ResTech and international standards leveraging on RegTech and SupTech discussions. In this regard, rather than industry players, both regulators and resolution authorities are the best placed to drive innovation.
